# Hemivertebra with pathogenic microdeletion of chromosome 9

**DOI:** 10.1002/ccr3.8750

**Published:** 2024-04-07

**Authors:** Yeshey Dorjey, Tashi Gyeltshen

**Affiliations:** ^1^ Department of Obstetrics and Gynecology, Phuentsholing General Hospital Chukha Bhutan; ^2^ Department of Radioimaging and Diagnosis, Jigme Dorji Wangchuk National Referral Hospital Thimphu Bhutan

**Keywords:** abnormal karyotype, chromosomal deletion, congenital abnormalities, spine

## Abstract

Hemivertebra is a rare congenital abnormality of the spinal column. Hemivertebra with other structural and cytogenetic abnormalities are reported. The prognosis is favorable with partial hemivertebra and with a single spinal defect as compared to a defect involving full segments and affecting different levels of the spines. The perinatal outcome is obscured when it is associated with other syndromes or cytogenetic abnormality. It is imperative to do serial thorough anatomical ultrasound scanning and to screen for chromosomal abnormality when hemivertebra is detected during pregnancy.

## 1 INTRODUCTION

Hemivertebra is a rare congenital abnormality of the spinal column where only half of the vertebra develops.^1^ The incidence of hemivertebra is over 1 per 10,000 live births.^2^ The exact etiology is elusive; however, many causes are being postulated. The most common cause reported is a developmental abnormality in the spinal column.^3^ The vertebra develops from mesoderm during gastrulation, where mesodermal cells divide and form a sclerotome. Sclerotome cells unit together to form fetal vertebrae which start at 6 weeks and are completed by 13 weeks of gestation.^4^ There are two primary ossification centers located each on the dorsal and ventral areas of the developing vertebrae. These ossification centers spread out and form the vertebral body and vertebral process from dorsal and ventral ossification centers, respectively.^5^ When one or both ossification centers are abnormal, or absent; abnormal vertebrae develop which are termed hemivertebrae. Other etiology postulated are abnormal intervertebral segmental arteries, a mutation in HOX genes, and exposure to teratogens during the pregnancy period.^6^ Hemivertebrae are classified depending on the segmentation as fully segmented, partially segmented, and unsegmented hemivertebrae. Fully segmented hemivertebra is the most common and has deleterious clinical significance as compared to the other two types.^7^ The overall prognosis of hemivertebra depends on; associated other abnormalities and associated with other syndromes .^6^, ^8^ In the literature, few cases of hemivertebra were reported to have structural and cytogenetic abnormalities which includes tetrasomy 4q, mosaic trisomy 4, mosaic trisomy 7, mosaic trisomy 9, mosaic trisomy 18q, mosaic trisomy 18; trisomy 7, trisomy 15q with monosomy 6q, partial trisomy 22, duplication of 2p, 4p‐ deletion, 17p deletion, 18p deletion, 18q22.2 deletion, 22q13.3 deletion, and ring chromosome 21.^9^, ^10^ However, pathogenic microdeletion of chromosome 9 is not reported in the literature. This write‐up is to report a pregnant mother at 19 weeks gestation with hemivertebra with a pathogenic microdeletion of chromosome 9.

## 2 CASE PRESENTATION

A 24‐year‐old, primigravida at 19 weeks gestation presented for routine anomaly scanning. She did not have any other medical disorders, no family history of spinal defect, syndrome, or genetic disorders. She has no exposure to toxic substances during pregnancy, and all the clinical parameters are within normal limits. Her blood group is A, rhesus positive. The screening for Down's syndrome with the triple test was negative, and all the serological tests were negative.

## 3 METHODS (INVESTIGATIONS)

A detailed anomaly scan was performed and noted normal findings in the fetal brain and neck.

On the facial profile, nasal bone was present, orbits were normal, lips and palate were seen, and there was no cleft lip or cleft palate.

Fetal lungs appeared normal, and fetal heart and fetal echocardiography findings were normal. The fetal abdomen appeared normal, there was no thoracoabdominal wall defect. Fetal kidneys and urinary bladder appeared normal with two umbilical arteries present.

The extremities and the limbs of the fetus appeared normal.

**FIGURE 1:**
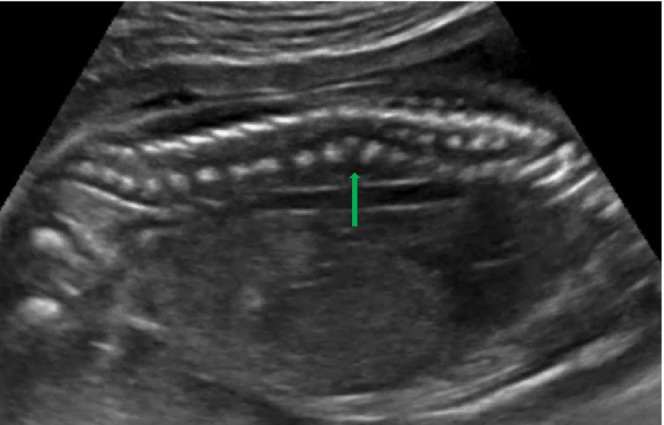
Ultrasound image showing angulation in fetal spine in sagittal plane (green arrow).

**FIGURE 2:**
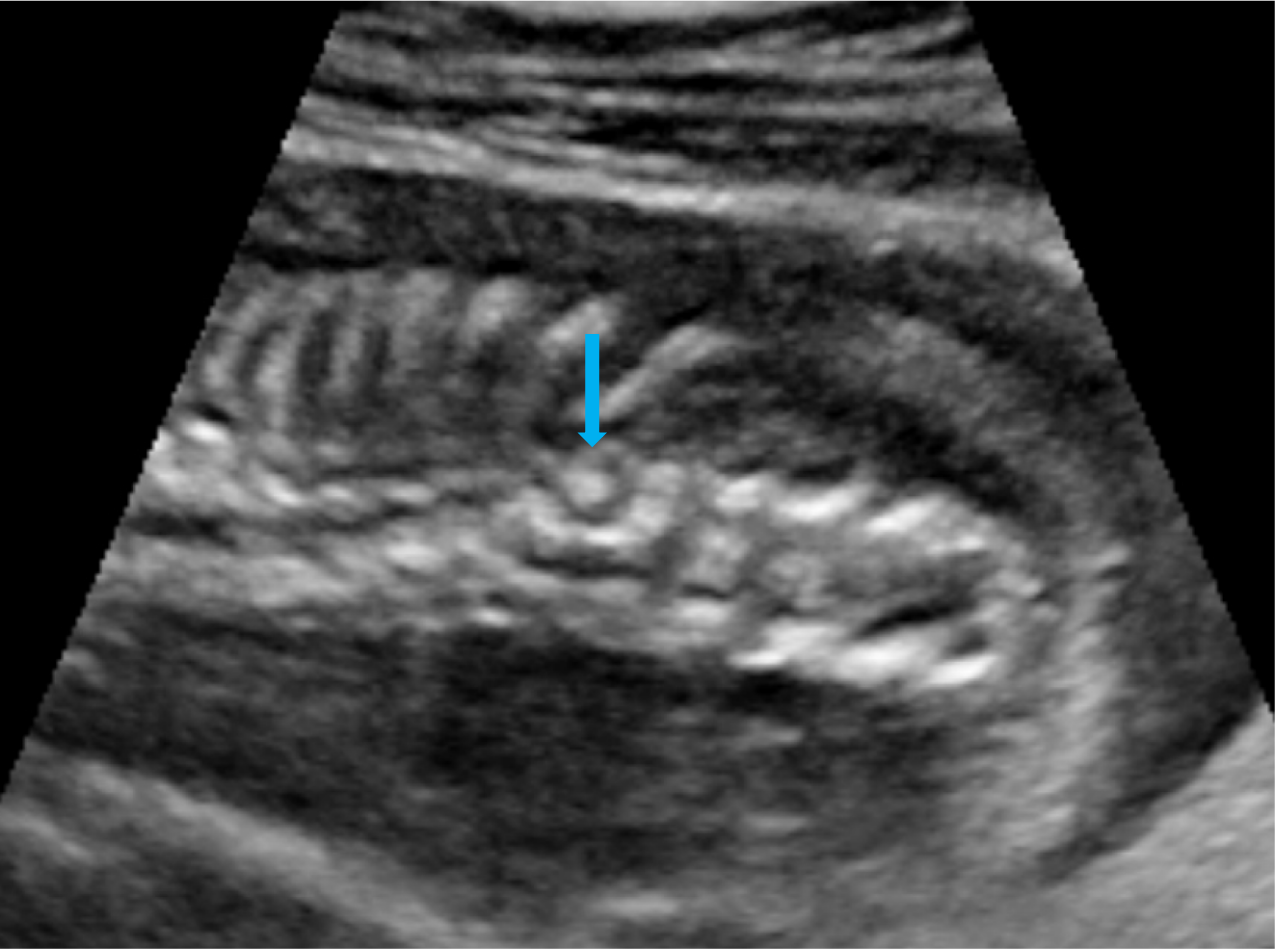
Ultrasound image of fetal spine in the coronal plane showing wedge‐shaped vertebra (blue arrow).

**FIGURE 3:**
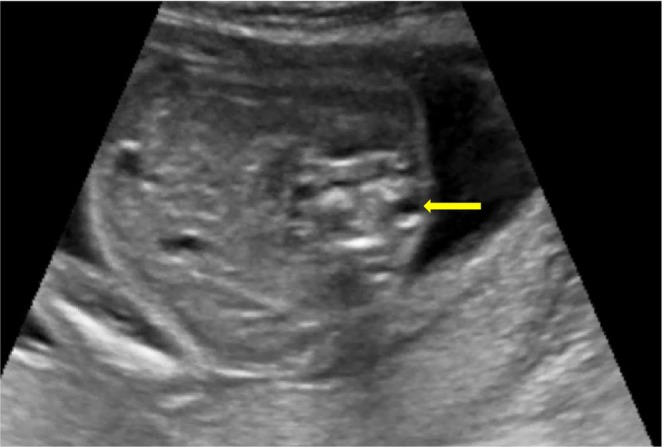
Ultrasound image of fetal spine in axial view showing the absence of a right segment of a vertebra (yellow arrow).

On scanning of the fetal spine, in the sagittal plane, abnormal angulation of the spine was present at the T11–T12 region (Figure 1), and in the coronal plane, the vertebra appeared wedge shaped with an absence of the right spinal segment at the T11 region (Figure 2). An axial view of the spine showed the absence of the right side of the vertebral segment (Figure 3).

After the complete anomaly scanning, the diagnosis was primigravida 19 weeks gestation with right hemivertebra. The patient was counseled on the risk of associated anomalies, genetic syndromes, aneuploidy, and the prognosis of hemivertebra. Amniocentesis was performed and karyotype analysis showed a normal, male karyotype (46, XY). The same amniotic fluid was used for chromosomal microarray analysis (CMA) and the report showed “pathogenic” microdeletion of chromosome 9 (9q22.1q22.32). The final diagnosis was primigravida 19 weeks gestation with right hemivertebra with pathogenic microdeletion of chromosome 9 (9q22.1q22.32).

The case was discussed with the pediatric surgeon, neonatologist, and orthopedic surgeon, and decided to continue the pregnancy. Pregnancy was followed up more frequently with serial ultrasound scanning for monitoring fetal growth, deterioration of hemivertebra, and survey for other associated abnormalities.

## 4 OUTCOME

**FIGURE 4:**
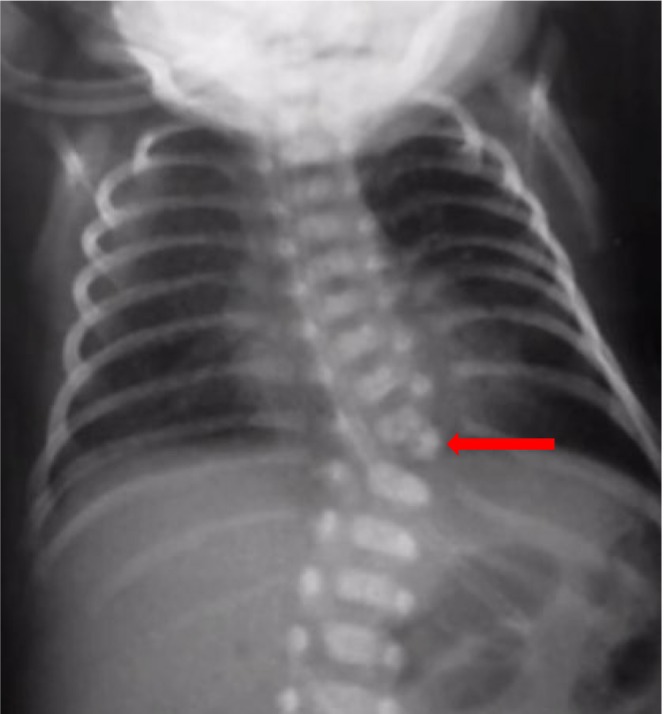
X‐ray of thoraco‐lumbar spines of newborn showing hemivertebra at T11 level (red arrow).

At 39 weeks and 5 days of gestation, patient went into spontaneous labor and delivered vaginally a male baby weighting 3100 g, with an Apgar score of 9 in first minute and 10 in 5 minutes. At birth, no other birth defect or anomaly was present except for mild angulation of spine was noticed at thoracolumbar level. X‐ray of spine was performed and showed hemivertebra at T11 level (Figure 4). The baby was assessed for associated skeletal, cardiac, renal, and gastrointestinal anomalies and showed normal finding. The orthopedic surgeon is consulted, and baby is being followed at the Orthopedics Department to look for progression of scoliosis.

## 5 DISCUSSION

This write‐up reports a rare case of hemivertebra with pathogenic microdeletion of chromosome 9. As per the existing evidence in the literature, the association of hemivertebra with chromosomal abnormality is not established. However, a few studies on hemivertebra have reported to have cytogenic abnormalities and fewer had structural anomalies.^9^, ^10^, ^11^ In the current case, karyotyping performed on the amniotic fluid showed normal karyotype (46, XY); however, the CMA report showed pathogenic microdeletion of chromosome 9 (9q22.1q22.32) which was not reported in the literature.

Hemivertebra is reported to be rarely associated with other abnormalities like central nervous, cardiovascular, gastrointestinal, genitourinary, and musculoskeletal systems.^8^, ^12^

The exact cause of the hemivertebra remains unknown; however, developmental abnormality of the vertebral column, abnormal intersegmental arteries of the vertebra, mutation of HOX genes, and exposure to teratogens were the postulated etiology.^6^

The hemivertebra causes spinal angulation deformities like scoliosis, kyphosis, and lordosis. The angulation deformity is more severe with the fully segmented hemivertebra as compared to partially segmented and non‐segmented types. In the fully segmented hemivertebra, the ossification centers are left open on both dorsal and ventral surfaces and the vertebra keeps growing in both directions adding more curvature to the angulated spines.^5^

The overall prognosis of hemivertebra depends on the types of hemivertebra, associated other abnormalities, associated syndromes, and aneuploidy.^6^ Prognosis is favorable with single hemivertebra as compared to multiple hemivertebrae.^13^ The current case was an isolated right‐sided hemivertebra and the prognosis is likely favorable although pathogenic microdeletion of chromosome 9 was detected.

Prenatal high‐resolution real‐time ultrasonography is sufficient to diagnose hemivertebra, however, when associated with complex anomalies and in doubtful cases, fetal MRI can be performed for further confirmation. In addition, 3D imaging tools can be used to precisely detect spine abnormalities.^14^, ^15^ In the index case, hemivertebra was diagnosed during the prenatal period with ultrasound scanning which corresponded with the x‐ray findings performed during the postnatal period.

There are a few cases of hemivertebrae who underwent termination of pregnancy. Termination can be offered to pregnant mothers with multiple hemivertebrae or associated with lethal anomalies or syndromes. Termination of pregnancy is offered only after thorough counseling of couples and after getting informed consent.

The management of the hemivertebra requires a multidisciplinary approach involving a maternal‐fetal medicine specialist, neonatologist, pediatric surgeon, and orthopedic surgeon.^13^ There is no intrauterine therapy or intervention available for the hemivertebra, most of the cases underwent surgical intervention in the postnatal period.

## 6 CONCLUSION

A fetus with hemivertebra diagnosed during the prenatal period had a pathogenic microdeletion on chromosome 9 (9q22.1q22.32). After a diagnosis of hemivertebra, a thorough assessment for other associated structural or cytogenetic abnormalities is required. This will help a couple to make an informed decision to terminate or to continue the pregnancy. A complex hemivertebra with lethal anomalies may require termination of pregnancy. This re‐emphasizes the importance of performing karyotyping including chromosomal microarray analysis for the fetuses with hemivertebra which may help in planning for the management.

## AUTHOR CONTRIBUTIONS

**Yeshey Dorjey:** Conceptualization; data curation; formal analysis; investigation; methodology; project administration; resources; supervision; validation; visualization. **Tashi Gyeltshen:** Conceptualization; methodology; resources; validation; visualization; writing – original draft; writing – review and editing.

## FUNDING INFORMATION

No funds are involved in writing this article.

## CONFLICT OF INTEREST STATEMENT

The authors have no conflicts of interest.

## CONSENT

Informed written consent has been obtained from the patient to collect the case history, collect the data, take the pictures, and write a case report for publication in medical journals.

## Data Availability

The data that support this writing are available from the corresponding author upon reasonable request.
